# Evaluating Patient Experience with Integrated Virtual Care (IVC), a Hybrid Primary Care Model in Rural Ontario, Canada: A Cross-Sectional Survey

**DOI:** 10.1177/21501319251345741

**Published:** 2025-06-29

**Authors:** Samantha Buchanan, Shawna Cronin, Antoine St-Amant, Jonathan Fitzsimon

**Affiliations:** 1 Department of Family Medicine, University of Ottawa, Ottawa, ON, Canada; 2 Institut du Savoir Montfort, Ottawa, ON, Canada

**Keywords:** patient satisfaction, patient experience, primary care, virtual care, team-based care

## Abstract

**Introduction:**

Canada faces a primary care crisis, especially in rural regions. In Ontario, the innovative, Integrated Virtual Care (IVC) program is a hybrid care model that enrolls patients with a family physician working predominantly remotely, while also embedded in a local Family Health Team, blending virtual and in-person care.

**Objective:**

To evaluate the experience of patients enrolled in IVC.

**Methods:**

We conducted a cross-sectional survey in a rural region in eastern Ontario, Canada. Participants included individuals enrolled in IVC for a minimum of 6 months. Primary outcome measures focused on patient experience with IVC, including satisfaction, access, self-reported health, and healthcare utilization. We also examined representativeness of survey respondents.

**Results:**

198 of 790 patients responded (response rate of 25.1%). Overall satisfaction was high, with 85% reporting being very satisfied or satisfied with IVC. Experiencing issues with virtual care was significantly associated with satisfaction. Survey respondents were generally older, Caucasian, and higher users of the healthcare system compared to a group of all those eligible to complete the survey.

**Conclusion:**

This study indicates high patient satisfaction with IVC among survey respondents. These insights can inform the expansion of innovative hybrid care models to meet the needs of underserved, rural populations.

## Introduction

In Canada, primary care attachment (or enrollment, empanelment) ensures that a designated primary care provider (PCP) – typically a family physician (FP) or, in some cases, a nurse practitioner (NP) – is accountable for coordinating and managing a patient’s care. However, primary care is in crisis, with 22% of Canadians lacking a PCP.^1^ This challenge extends beyond Canada, as the United States is projected to require an additional 60,000 PCPs by 2040 to meet the demands of an aging population.^2^ Rural and remote communities face disproportionate barriers to primary care access, with only 8% of physicians practicing in these regions despite 18% of the population residing there.^3^ Being without a regular PCP can lead to fragmentated or absent primary care, leading to increased reliance on emergency departments (EDs), poorer health outcomes, and higher mortality rates.^4-7^

To address the challenge of insufficient primary care attachment, the Integrated Virtual Care (IVC) program was launched in fall 2021 in Renfrew County, Ontario.^8^ This innovative, hybrid model delivers comprehensive primary care under FP leadership, aligning with the Patient’s Medical Home framework – Canada’s vision for high-quality primary care, as outlined by the College of Family Physicians of Canada^9^ (Box 1). This framework emphasizes comprehensive, team-based primary care with FP leadership, accessibility, and continuity of care.^10^ IVC offers both in-person and virtual care options that patients can access from a local clinic or their home. Previously unattached patients are enrolled to a named FP who coordinates all aspects of their care, while being embedded within a local interdisciplinary Family Health Team (FHT). Care from FPs is delivered predominantly through virtual modalities, including secure messaging, telephone, and video appointments. In-person care is delivered by locally based team members, including FPs, NPs, community paramedics, and other allied health professionals (AHPs) within the FHT.^8^

Box 1. **What Is the Patient’s Medical Home?**

The Patient’s Medical Home (PMH) is a framework proposed by the College of Family Physicians of Canada to define a vision for family practices in Canada. It emphasizes patient-centered care, community adaptiveness, and interprofessional collaboration with FP leadership. The PMH envisions care that is readily accessible, tailored to patients’ needs, delivered across all life stages, and seamlessly integrated with other health and community services.^11^

Leveraging virtual care alongside in-person services expands the pool of available providers by enabling access to a province-wide pool of FPs, rather than relying solely on those available locally. Virtual care also offers key advantages, including accessibility, convenience, flexibility, and reduced risk of disease transmission for both patients and providers.^12,13^ Moreover, existing evidence supports its general safety and clinical efficacy.^14,15^ However, virtual care may feel impersonal or “mechanical” to some patients and can be challenging for individuals with limited digitally literacy, poor internet access, or health issues requiring physical examination. These limitations can result in fragmented care or necessitate additional in-person follow-up, particularly for issues that might have been resolved in a single visit with an in-person family physician.^16,17^

Previous research on patient experience with virtual care indicates a high satisfaction across various modalities, including telephone, video, and asynchronous messaging.^18-21^ An early assessment of IVC found similar satisfaction levels to those reported in traditional, in-person primary care, with 90% of patients expressing satisfaction.^22^ When incorporated into traditional, in-person primary care practice, patients report a high level of satisfaction with virtual visits.^23^ However, there has been limited evaluation of programs that predominantly rely on virtual modalities for the delivery of comprehensive primary care (Box 2). This study aims to assess the experience and satisfaction of patients enrolled in IVC, a team-based, hybrid model of primary care in rural Ontario, Canada.

Box 2. **From Triple to Quintuple Aim: A Framework for Health System Improvement.**

In 2008, Berwick et al^24^ of the Institute for Healthcare Improvement proposed that improving health systems required the simultaneous pursuit of 3 goals: enhancing the experience of care, improving population health, and reducing per capita healthcare costs. This framework, known as the Triple Aim, was later expanded to include a fourth aim – improving the experience of healthcare providers^25^ – and more recently, a fifth: advancing health equity.^26^ Together, these 5 goals form the Quintuple Aim, which now guides the design and evaluation of healthcare models.

## Methods

### Study Design and Setting

In this cross-sectional survey, we used a non-probability convenience sampling approach, inviting all IVC patients aged 18 and older who had been receiving care in the program for at least 6 months as of October 2022 and had attended at least 1 physician visit during that period. The survey was conducted between October and December 2022. This study adheres to the Checklist for Reporting of Survey Studies (CROSS) guidelines.^27^

Renfrew County (population 106,365) is located in eastern Ontario and is predominantly rural, spanning 7600 square kilometers. It has some of the highest rates of chronic physical and mental illness in the province, compounded by lower-than-average socioeconomic status.^28^ The region also experiences high unattachment rates, with data indicating that approximately 20% of individuals in Renfrew County are not attached to a FP.^29^ The Petawawa Centennial Family Health Centre (PCFHC), the base site of IVC, is located in the town of Petawawa, population 14 382.^28^ The PCFHC operates with an interdisciplinary team, including physicians, social workers, a pharmacist, a registered dietitian, and nurses. The clinic serves a roster of more than 8000 patients, primarily from Petawawa. At the time of this study, 1595 patients were rostered to IVC.

### Survey

A 3 part, anonymous survey containing 33 questions was developed, including an introduction with screening questions, a section on satisfaction with IVC, and a section on respondents’ socio-demographic and health characteristics (Supplementary File 1). The satisfaction section assessed the following key dimensions: access to care, experiences with virtual modalities, patient-centered care and overall experience, adapting items from previously validated surveys (Supplementary File 1).^30-32^ To assess trust as a fundamental component of the patient-provider relationship, the survey included items from the validated Trust in Physician scale.^20,32^ Virtual care-related questions were informed by the Association of Family Health Teams of Ontario Virtual Care Experience survey.^31^ Sociodemographic and health-related questions were guided by recent literature.^33^ The survey was developed and tested with healthcare leaders from the PCFHC and the Ottawa Valley Ontario Health Team. It was hosted on OceanMD, a secure platform used by IVC providers to communicate with patients, ensuring familiarity and ease of access.^34^ OceanMD has been successfully utilized in previous surveys from PCFHC and a previous study by our group.^22^

### Data Collection

Between October and December 2022, all eligible IVC patients were invited to participate in the survey via an initial email sent to the address on file in the IVC patient records, followed by 3 reminders over the 2-month period, thanking respondents and encouraging non-respondents to complete the survey. Data from the electronic medical records (EMRs) of all individuals who received the survey were used to assess representativeness of survey respondents. Comparison variables included: age, sex, NP visit count, and number of visits with AHPs. To encourage participation, survey respondents were entered into a draw for one of two 100$ gift cards.

### Data Analysis

We first assessed survey representativeness by comparing respondents to the sampling frame of all eligible patients invited to participate in the survey using chi-square tests to examine differences in sex, age, and the number of in-person visits with NPs and AHPs. In-person NP visits serve as a proxy for referrals following virtual appointments with FPs, indicating a need for in-person support. Within the IVC model, this follow-up care is primarily delivered by group NPs, although group FPs are also available on-site. Frequency analysis was conducted for all survey questions, categorizing responses according to their respective 5-point Likert options.^35^ Finally, we analyzed the correlation between overall satisfaction levels (“Very Satisfied/Satisfied,” “Neither Satisfied nor Dissatisfied,” and “Very Dissatisfied/Dissatisfied”) and gender, age, income, overall health, reported virtual care issues, and appointments with NPs and AHPs, using chi-square tests and Fisher’s exact tests where applicable. A 2-tailed *P*-value <.05 was considered statistically significant. Only 1 question had missing responses (<2% of participants). Answers to that question were omitted from the analysis, without excluding the entire participant record. All analyses were conducted using SAS v. 9.4 and R v. 4.3.0.^36,37^

## Results

The survey was distributed to all eligible IVC patients (n = 790) aged 18 years and older who were enrolled in the program between November 2021 (inception) and June 2022. A total of 203 individuals responded. After excluding those who did not meet the screening criteria, 198 respondents were included in the final analysis, with a response rate of 25.1%.

When comparing the 198 survey respondents to the 790 eligible IVC patients, the proportion of men and women were similar. However, respondents were older, with a greater share aged 55 to 74 (41.9% vs 30.8%). Additionally, a larger proportion of survey respondents had accessed an AHP (44.4% vs 9.1%) or NP (41.9% vs 27.2%) (Table 1).

**Table 1.** Survey Respondents Compared to IVC Participants.

Variable	Category	Survey respondents (n = 198)	Survey sampling frame (n = 790)	*P*-value
Sex^a^	Female	123 (62.8)	519 (65.7)	.4518
	Male	73 (37.2)	271 (34.3)	
Age	18-34	28 (14.1)	212 (26.8)	.0030
	35-44	43 (21.7)	156 (19.8)	
	45-54	33 (16.7)	130 (16.5)	
	55-64	57 (28.8)	153 (19.4)	
	65-74	26 (13.1)	90 (11.4)	
	75+	11 (5.6)	49 (6.2)	
Allied health appointment	Yes	88 (44.4)	72 (9.1)	<.0001
Nurse practitioner appointment	Yes	83 (41.9)	215 (27.2)	<.0001

a Two survey respondents identified as “non-binary,” which accounts for the total not summing to 198.

Survey respondents were predominantly female (62.1%), white (86.9%), and preferred to receive care in English (97.5%). Despite the rural setting, 79.8% reported living within a 10-minute drive of the clinic, while only 3% resided more than 30 min away. Additionally, 16.2% of respondents reported experiencing difficulties paying bills at least some of the time (Table 2).

**Table 2.** Demographic and Health Characteristics of Survey Respondents (n = 198).

Variable	Category	Count (n)	Percentage
Gender	Male	73	36.87
	Female	123	62.12
	Non-binary	2	1.01
Age	18-34	28	14.14
	35-44	43	21.72
	45-54	33	16.67
	55-64	57	28.79
	65-74	26	13.13
	75 plus	11	5.56
Race	White	172	86.87
	First nation or metis	10	5.05
	Asian	4	2.02
	Black, middle eastern, mixed heritage	4	2.02
	Prefer not to answer	8	4.04
Sexual orientation	Heterosexual	183	92.42
	Bisexual, pansexual, or queer	6	3.03
	Prefer not to say	9	4.55
Self-rated overall health	Excellent	27	13.63
	Good	80	40.40
	Average	65	32.82
	Poor	22	11.11
	Very poor	4	2.02
Travel time to clinic	Less than 10 min	158	79.80
	10-29 min	31	15.66
	More than 30 min	6	3.03
	Missing	3	1.52
Difficulty paying bills?	No	150	75.76
	Sometimes	18	9.09
	Yes	14	7.07
	Prefer not to answer	16	8.08
Preferred language to receive care	English	193	97.47
	French	5	2.53

### Overall Satisfaction

Overall, 85.4% (n = 169) of respondents reported being very satisfied or satisfied with their care from IVC. Most respondents (69.7%, n = 138) rated their overall experience with their physician as excellent or very good, while only 4.6% reported a poor experience (Table 3). Among the 88 patients who had at least 1 appointment with an AHP, 96.5% (n = 85) reported an excellent, very good, or good experience. Similarly, among the 83 patients who had at least 1 appointment with a NP, 89.2% (n = 74) reported a positive experience.

**Table 3.** Overall Satisfaction (n = 198).

Variable	Category	Count (n)	Percentage
Overall experience with physician	Excellent	89	44.95
	Very good	49	24.75
	Good	40	20.20
	Fair	11	5.56
	Poor	9	4.55
Received conflicting info from health team?	Yes	15	7.58
	Not sure	8	4.04
	No	164	82.83
	N/A	11	5.56
Would you recommend IVC to a friend?	Very likely	120	60.61
	Somewhat likely	36	18.18
	Neutral	25	12.63
	Somewhat unlikely	10	5.05
	Very unlikely	7	3.54
Overall level of satisfaction with IVC	Very satisfied	98	49.49
	Satisfied	71	35.86
	Neither satisfied nor dissatisfied	10	5.05
	Dissatisfied	14	7.07
	Very dissatisfied	5	2.53

In total, 77.3% (n = 153) were very satisfied or satisfied with their ability to book an appointment on their preferred day. When assessing factors associated with satisfaction, the only variable significantly correlated with satisfaction was whether respondents reported issues using virtual technology (*P* < .0001). Other factors, including age, gender, and appointment modality, were not significantly associated with satisfaction (Table 4).

**Table 4.** Characteristics of Participants by Satisfaction Level.

Variable	Category	Very satisfied/satisfied	Neither	Very dissatisfied/dissatisfied	Total	*P*-value
		n (%)	n (%)	n (%)	n (%)	
		169 (85.35)	10 (5.05)	19 (9.60)	198	
Gender^a^	Female	106 (63.50)	7 (70.00)	10 (58.60)	123 (62.10)	.5785
	Male	61 (36.63)	3 (30.00)	9 (47.37)	73 (36.87)	
	Non-binary	2 (1.18)	0 (0)	0 (0)	2 (1.01)	
Age	18-34	26 (15.38)	0(0)	2 (10.53)	28 (14.14)	.3496
	35-44	36 (21.30)	2 (20.00)	5 (26.32)	43 (21.72)	
	45-54	31 (18.34)	0 (0)	2 (10.53)	33 (16.67)	
	55-64	44 (26.04)	5 (50.0)	8 (42.11)	57 (28.79)	
	65+	32 (18.93)	3 (30.0)	2 (10.53)	37 (18.69)	
Difficulty paying bills at the end of the month	Yes	13 (7.69)	0 (0)	1 (5.26)	14 (7.07)	.4572
	Sometimes	16 (9.47)	0 (0)	2 (10.53)	18 (9.09)	
	No	129 (76.33)	8 (80.0)	13 (68.42)	150 (75.76)	
	Prefer not to answer	11 (6.51)	2 (20.0)	3 (15.79)	16 (8.08)	
Overall health	Excellent	26 (13.13)	0 (0)	1 (5.26)	27 (13.64)	.0706
	Good	71 (42.01)	4 (40.0)	5 (26.32)	80 (40.40)	
	Average	54 (31.95)	4 (40.0)	7 (36.84)	65 (32.83)	
	Poor	16 (9.47)	2 (20.0)	4 (21.05)	22 (11.11)	
	Very poor	2 (1.18)	0 (0)	2 (10.53)	4 (2.02)	
Reported no issues with virtual care	Yes	143 (84.62)	4 (40.0)	5 (26.32)	152 (76.77)	<.0001
	No	26 (15.38)	6 (60.0)	14 (73.68)	46 (23.23)	
Had appt with NP	Yes	73 (43.20)	3 (30.0)	7 (36.84)	83 (41.92)	.9419
	No	96 (56.80)	6 (60.0)	11 (57.89)	103 (52.02)	
	Unsure	10 (5.92)	1 (10.0)	1 (5.26)	12 (6.06)	
Had appt with AHP	Yes	76 (44.97)	3 (30.0)	9 (47.37)	88 (44.44)	.7195
	No	77 (45.56)	5 (50.0)	9 (47.37)	91 (45.96)	
	Unsure	16 (9.47)	2 (20.0)	1 (5.26)	19 (9.60)	

Abbreviations: AHP, allied health professional; NP, nurse practitioner.

a Non-binary respondents were excluded due to limited sample size; *P*-values are from Pearson’s Chi-square tests. A sensitivity analysis using Fisher’s Exact Test with Monte Carlo simulation yielded similar values.

### Trust and Care from Physician

Patients reported high levels of trust in their physician across all survey measures. The majority strongly agreed or agreed that they had the opportunity to ask questions (85.9%, n = 170), were involved in treatment decisions (81.3%, n = 161) and trusted their physician enough to follow their advice (71.5%, n = 142) (Table 5).

**Table 5.** Trust in Physician (n = 198).

Variable	Category	Count (n)	Percentage
Opportunity to ask questions	Strongly agree	83	41.92
	Agree	87	43.94
	Neither agree nor disagree	10	5.05
	Disagree	8	4.04
	Strongly disagree	2	1.01
	N/A	8	4.04
Involved in treatment decisions	Strongly agree	80	40.40
	Agree	81	40.91
	Neither agree nor disagree	20	10.10
	Disagree	10	5.05
	Strongly disagree	3	1.52
	N/A	4	2.02
Spent enough time with me	Strongly agree	74	37.37
	Agree	88	44.44
	Neither agree nor disagree	13	6.57
	Disagree	15	7.58
	Strongly disagree	6	3.03
	N/A	2	1.01
Trust and follow advice of physician	Strongly agree	66	33.33
	Agree	76	38.38
	Neither agree nor disagree	38	19.19
	Disagree	10	5.05
	Strongly disagree	6	3.03
	N/A	2	1.01
Trust in physician’s judgments about care	Strongly agree	77	38.89
	Agree	77	38.89
	Neither agree nor disagree	29	14.65
	Disagree	9	4.55
	Strongly disagree	4	2.02
	N/A	2	1.01
Trust physician to tell if mistake was made	Strongly agree	66	33.33
	Agree	83	41.92
	Neither agree nor disagree	31	15.66
	Disagree	8	4.04
	Strongly disagree	6	3.03
	N/A	4	2.02

### Experience with Virtual Care

**Figure 1.**
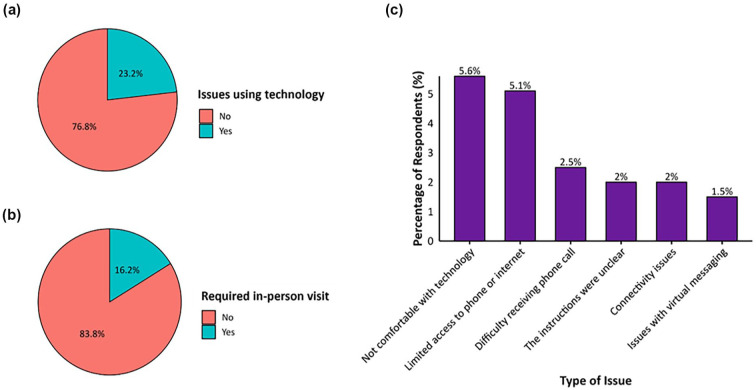
Proportions of respondents who (a) agreed or strongly agreed with the statement, “The technology was easy to use” during their most recent virtual appointment with IVC; (b) reported requiring an in-person follow-up visit (yes/no) after their most recent IVC virtual appointment; and (c) identified specific challenges encountered during their most recent IVC virtual visit.

Patients’ experience and challenges with using virtual care are summarized in Table 6 and Figure 1. The majority (76.8%, n = 152) reported no issues using technology, and 81.8% (n = 162) felt their health concern was appropriately addressed through virtual care. Additionally, 80.3% indicated that virtual care saved them money, while 70.7%, reported that it saved them time. Lastly, only 16.2% of patients reported that their health issue required an in-person visit, while 5.6% indicated discomfort with technology, and 5.1% faced limited access to phone or internet.

**Table 6.** Experience with Virtual Care (n = 198).

Variable	Category	Count (n)	Percentage
The technology was easy to use	Strongly agree	65	32.83
	Agree	83	41.92
	Neither agree nor disagree	17	8.59
	Disagree	3	1.52
	Strongly disagree	0	0
	N/A	30	15.15
The level of privacy was appropriate	Strongly agree	95	47.98
	Agree	85	42.93
	Neither agree nor disagree	15	7.58
	Disagree	0	0
	Strongly disagree	0	0
	N/A	3	1.52
I felt safe during my appointment	Strongly agree	105	53.03
	Agree	78	39.39
	Neither agree nor disagree	12	6.06
	Disagree	2	1.01
	Strongly disagree	0	0
	N/A	1	0.51
Communicate health issue	Strongly agree	77	38.89
	Agree	62	31.31
	Neither agree nor disagree	25	12.63
	Disagree	28	14.14
	Strongly disagree	4	2.02
	N/A	2	1.01
Virtual care saved me time	Strongly agree	93	46.97
	Agree	66	33.33
	Neither agree nor disagree	21	10.61
	Disagree	12	6.06
	Strongly disagree	3	1.52
	N/A	3	1.52
Virtual care saved me money	Strongly agree	72	36.36
	Agree	68	34.34
	Neither agree nor disagree	34	17.17
	Disagree	14	7.07
	Strongly disagree	3	1.52
	N/A	7	3.54
My health concern was addressed	Strongly agree	82	41.41
	Agree	80	40.40
	Neither agree nor disagree	14	7.07
	Disagree	12	6.06
	Strongly disagree	6	3.03
	N/A	4	2.02

## Discussion

We conducted a cross-sectional survey to assess patient experience and satisfaction with IVC, a hybrid program where patients are attached to a FP providing comprehensive care, primarily virtually, with in-person support. Results indicate high satisfaction across all survey components, regardless of whether the encounter was with a FP, NP, or AHP. The highest satisfaction rates (≥90%) were reported for in-person encounters with NPs and AHPs, consistent with previous research from our group and others, which suggests that patients view in-person support as a key component of hybrid care programs.^38-40^ High satisfaction across survey components reinforces our earlier findings, which showed consistently high patient satisfaction regardless of prior attachment status before joining IVC.^22^

IVC is unique in providing comprehensive primary care predominantly through virtual care, alongside a broad range of in-person options. Most studies on virtual care have focused on acute, episodic care. For example, a recent study on the Renfrew County Virtual Triage and Assessment Centre (VTAC) – the broader program under which the IVC serves as the attachment arm – found an 89% satisfaction rate with virtual care encounters. The overall satisfaction rate with IVC was only slightly lower than this, at 85%, despite IVC’s mandate to provide the full scope of comprehensive primary care. This small difference may reflect VTAC’s maturity or suggest that some patients are more receptive to receiving virtual care for single, acute issues than in the context of a long-term, patient-provider relationship.^38^ Other studies assessing virtual primary care options have also reported patient satisfaction rates in a similar range (82–91%).^19,20^

Agreement with survey statements regarding trust in IVC physicians ranged from 72% to 78%, comparing favorably to other surveys where trust in primary care physicians varies between 59% and 90%, depending on factors such as age, income, and race.^41^ Trust has been linked to better continuity of care, higher patient satisfaction, and greater patient involvement.^42^ Additionally, the patient-provider relationship is a key determinant of satisfaction in virtual care programs.^43,44^ The overall satisfaction with IVC and trust in IVC physicians demonstrated in this study reflect an openness to virtual and hybrid care, consistent with findings from Canada and the U.S. For example, Stamenova et al^45^ reported that 99% of patients would use virtual visits again.^14^ Trust in physicians and hospitals declined across all socio-demographic groups during the COVID-19 pandemic. For example, a recent U.S. survey reported that trust dropped from over 70% in April 2020 to as low as 40% in January 2024.^46^ Despite this broader decline, participants in this study maintained generally high levels of trust in their care providers.

While virtual care may contribute to the “digital divide”, concerns about digital literacy may be overstated, particularly since most IVC virtual appointments are phone-based.^16,47^ In our study, only 6% of patients reported discomfort with technology. Virtual care can also help reduce travel burdens, a major barrier for some patients. Recent estimates suggest that some individuals spend up to 23 h per month on healthcare-related activities.^48^ In our study, over 70% of respondents reported time and cost savings from virtual care. Additionally, although high-speed internet access can be a barrier to virtual care, particularly in rural areas,^49^ this is unlikely to be a major issue for IVC, as most encounters occurred via phone, broadband access is available in many parts of the region,^50^ and IVC patients can attend their local clinic where staff can set up and assist a video encounter from the clinic between the patient and their physician.

The IVC program and this patient experience evaluation are set in a rural area with limited primary care options and a significant shortage of PCPs. Virtual care has been proposed as part of the solution to improve access in rural regions by expanding the pool of available physicians beyond the local area, potentially reducing ED use and minimizing the need for long-distance travel to clinics.^51^ An early clinical evaluation of IVC suggests that many patients who were overdue for preventive care became up-to-date after enrollment, which may have helped prevent them from becoming high users of healthcare.^8^ Our findings indicate that most IVC respondents live within a 10-minute drive of the clinic, suggesting that travel distance is not a major barrier for this group.

As part of an ongoing evaluation of IVC within the quintuple aim framework of quality improvement,^26^ the program has undergone early assessments of its patient and clinical impact.^8,22^ As IVC continues to expand – enrolling 6,647 patients across 3 sites as of February 2025, a more than 4-fold increase since this survey was conducted – A detailed clinical and economic evaluation of IVC is currently underway using population-level health data, along with a study examining the experiences of clinical and non-clinical IVC staff. Regarding patient experience, an in-depth qualitative evaluation using interviews could add to the findings of this study by providing deeper insights into this innovative care model and identifying opportunities for improvement.

This study provides valuable insights into the patient experience with IVC using a comprehensive survey. However, several limitations must be acknowledged. A key limitation relates to the online survey itself, which introduces self-selection bias.^52^ Nonetheless, this method was well-suited to the IVC context, as patients are familiar with OceanMD – the platform routinely used for communication from PCFHC. This familiarity may explain the relatively higher response rate (25%) compared to previous work using Microsoft Forms (<15%).^38^ Still, the response rate remains modest, introducing non-response bias and limiting generalizability to the broader IVC population. This may be partly due to the survey’s length, which prioritizes comprehensiveness but may have discouraged participation. Pilot testing with IVC patients or involving patient partners could have improved survey flow and engagement. Furthermore, respondents differed from the overall IVC population – they were older, predominantly Caucasian, and had more visits with NPs and AHPs, modalities generally associated with higher satisfaction – suggesting that findings should be interpreted with caution. Because the survey was anonymous, we could not identify which patients responded, and comparisons were made to all IVC patients, potentially underestimating representativeness differences.

Our study does not fully capture the experiences of younger adults (particularly given the exclusion of those under 18) or those with lower healthcare utilization. Lastly, as this study was conducted in rural Canada within a publicly funded healthcare system, the findings may not be fully applicable to other healthcare systems or urban settings. These limitations should be considered when interpreting the validity and generalizability of our results.

## Conclusions

This study shows that among a select group of IVC patients – an innovative hybrid model of in-person and virtual care through which patients receive comprehensive, team-based primary care – there are high levels of satisfaction, strong physician trust, and a positive overall experience. These findings support the potential of models like IVC to address FP shortages, particularly in rural areas.

## Supplemental Material

sj-docx-1-jpc-10.1177_21501319251345741 – Supplemental material for Evaluating Patient Experience with Integrated Virtual Care (IVC), a Hybrid Primary Care Model in Rural Ontario, Canada: A Cross-Sectional Survey Supplemental material, sj-docx-1-jpc-10.1177_21501319251345741 for Evaluating Patient Experience with Integrated Virtual Care (IVC), a Hybrid Primary Care Model in Rural Ontario, Canada: A Cross-Sectional Survey by Samantha Buchanan, Shawna Cronin, Antoine St-Amant and Jonathan Fitzsimon in Journal of Primary Care & Community Health

sj-docx-2-jpc-10.1177_21501319251345741 – Supplemental material for Evaluating Patient Experience with Integrated Virtual Care (IVC), a Hybrid Primary Care Model in Rural Ontario, Canada: A Cross-Sectional Survey Supplemental material, sj-docx-2-jpc-10.1177_21501319251345741 for Evaluating Patient Experience with Integrated Virtual Care (IVC), a Hybrid Primary Care Model in Rural Ontario, Canada: A Cross-Sectional Survey by Samantha Buchanan, Shawna Cronin, Antoine St-Amant and Jonathan Fitzsimon in Journal of Primary Care & Community Health
