# Supplementary material for: Evaluating Patient Experience with Integrated Virtual Care (IVC), a Hybrid Primary Care Model in Rural Ontario, Canada: A Cross-Sectional Survey
Source: J Prim Care Community Health. 2025 Jun 29;16:21501319251345741. doi: 10.1177/21501319251345741 (PMC12206991; doi:10.1177/21501319251345741)
Supplement: sj-docx-1-jpc-10.1177_21501319251345741 – Supplemental material for Evaluating Patient Experience with Integrated Virtual Care (IVC), a Hybrid Primary Care Model in Rural Ontario, Canada: A Cross-Sectional Survey [file sj-docx-1-jpc-10.1177_21501319251345741.docx]

**Supplementary File 1**

Evaluating Patient Experience with Integrated Virtual Care (IVC), a Hybrid Primary Care Model in Rural Ontario, Canada: A Cross-Sectional Survey

**Part A: Introduction**

*You are being invited to take part in this survey because you are a patient of an Integrated Virtual Health (IVC) family physician. Your responses to the questions will help us improve the care we provide. The survey will take approximately 5 minutes to complete. Participation in the survey is voluntary and all your responses to the survey questions are anonymous and will be kept confidential.*

*To participate in the IVC survey, there are several criteria that need to be met. Please answer the following questions truthfully.*

A1: I am at least 18 years of age

- 1. Yes
  2. No *(end survey)*

A2: have had at least one virtual encounter with my IVC family physician. Virtual encounters include phone calls, video calls, or secure messaging.

- 1. Yes
  2. No *(end survey)*

A3: I confirm that my responses to the questions listed above are true and correct to the best of my knowledge.

- 1. Yes
  2. No *(end survey)*

**Part B: Your Satisfaction with Integrated Virtual Care**

*Integrated Virtual Care (IVC) is a method of delivering comprehensive primary healthcare. As a patient of IVC, you may have virtual encounters with your family physician (phone call, video call, or secure messaging) but you may not meet them in-person. You can have in-clinic care from nurse practitioners and other allied health professionals at the Family Health Centre, as well as at-home care with a community paramedic or another member of the local family health team.*

***B1: Access to Care***

| *Very Dissatisfied* | *Dissatisfied* | *Neither Satisfied  nor Dissatisfied* | *Satisfied* | *Very Satisfied* | *N/A* |
| --- | --- | --- | --- | --- | --- |

How satisfied are you with the following?

B1A) Your ability to book an appointment on the day you wanted

B1B) The respect and curtesy shown by the Petawawa Centennial Family Health Center (PCFHC) clerks, receptionists, and medical secretaries

***B2: Your Virtual Care***

*The following questions refer to virtual appointments that you have had with your IVC family physician. Virtual appointments include phone calls, video calls, and/or secure messaging.*

| *Strongly Disagree* | *Disagree* | *Neither Agree  nor Disagree* | *Agree* | *Strongly Agree* | *N/A* |
| --- | --- | --- | --- | --- | --- |

Thinking about the most recent time you received care virtually with IVC, please tell us how much you agree or disagree with the following statements

B2A) The technology was easy to use

B2B) The level of privacy and confidentiality maintained during my appointment was appropriate

B2C) I felt safe during my virtual appointment

B2D) I was able communicate my health issue virtually as well as I would have in-person

B2E) Virtual care saved me time

B2F) Virtual care saved me money (e.g., by not having to pay for transportation/parking, care for dependents, not having to take time off work, etc.)

B2H) My health concern was addressed with the virtual visit

B2I) Thinking about your recent virtual appointment with your IVC family physician, have you experienced any of the following issues or concerns? (Select all that apply)

- No or limited access to computer/laptop/tablet or internet
- Not comfortable with technology
- Issues getting into the virtual appointment platform*
- Issues navigating the virtual appointment platform *
- My health issue required an in-person visit to address

***B3: Patient-Centered Care***

| *Strongly Disagree* | *Disagree* | *Neither Agree  nor Disagree* | *Agree* | *Strongly Agree* | *N/A* |
| --- | --- | --- | --- | --- | --- |

Thinking about your IVC family physician, how much do you agree with the following statements?

B3A) I had an opportunity to ask questions about recommended treatment

B3B) I was involved as much as I wanted to be in decisions about my care and treatment

B3C) My family physician spent enough time with me

B3D) I trust my family physician so much that I always try to follow their advice

B3E) I trust my family physician’s judgements about my medical care

B3F) I trust my physician to tell me if a mistake was made about my treatment

***B4: Overall Experience***

| *Very Dissatisfied* | *Dissatisfied* | *Neither Satisfied  nor Dissatisfied* | *Satisfied* | *Very Satisfied* | *N/A* |
| --- | --- | --- | --- | --- | --- |

*The following questions pertain to your overall experience with your IVC healthcare providers.*

*Family Physician*

B4A) Over the last 9 months, what type of encounters have you had with your family physician? (select all that apply)

- 1. Phone call
  2. Video call
  3. Secure messaging
  4. In-person visit

B4B) How would you rate your overall experience with your IVC family physician?

*Excellent, very good, good, fair, poor*

*Nurse practitioner*

B4C) Over the last 9 months, have you received care from a nurse practitioner?

*Yes/No*

B4D) How would you rate your overall experience with your nurse practitioner?

*Excellent, very good, good, fair, poor*

*Allied Health Team, including nurses, pharmacists, social workers, dietitians, and paramedics.*

B4E) Over the last 6 months, have you received care from a member of the allied health team?

*Yes/No*

B4F) How would you rate your overall experience with them?

*Excellent, very good, good, fair, poor*

B4G) In the last 9 months, was there ever a time when you received conflicting information about your healthcare from different members of your IVC healthcare providers such as your family physician, nurse practitioner, paramedic, dietician, or other members of the allied health team?

- 1. Yes
  2. No
  3. I don’t know

B4H) How likely are you to recommend IVC to your friends/family?

*Very likely, somewhat likely, neutral, somewhat unlikely, very unlikely*

B4I) Overall, how satisfied are you with the care you received with IVC?

*Very dissatisfied 🡪 very satisfied*

**Part D: About You**

C1) How old are you?

- 1. 18-24
  2. 25-34
  3. 35-44
  4. 45-54
  5. 55-64
  6. 65-74
  7. 75 or older

C2) Select the gender category you identify with:

1. Woman
2. Man
3. Two-Spirit
4. Trans woman
5. Trans man
6. Prefer not to answer
7. Another gender identity (please specify): ____

C3) Select the sexual orientation you identify with:

1. Heterosexual
2. Homosexual (Gay, Lesbian)
3. Bisexual
4. Queer
5. Other ____
6. Prefer not to answer

C4) Select the race(s)/ethnicity(ies) you identify with:

1. Asian – East (e.g., Chinese, Japanese, Korean)
2. Asian – South East (e.g., Malaysian, Filipino, Vietnamese)
3. Asian – South (e.g., Indian, Pakistani, Sri Lankan)
4. Indo-Caribbean (e.g., Guyanese with origins in India)
5. Middle Eastern/North African (e.g., Algerian, Iranian, Lebanese)
6. Black – Sub-Saharan African (e.g., Ghanian, Kenyan, Somali)
7. Black – North American (e.g., Canadian, American)
8. Black – Caribbean (e.g., Barbadian, Jamaican)
9. Latin American/Hispanic (e.g., Argentinean, Chilean, Salvadoran)
10. First Nations (Status/Non-Status Indian)
11. Inuk/Inuit
12. Métis
13. White – European (e.g., English, Italian, Russian)
14. White – North American (e.g., Canadian, American)
15. Mixed heritage (e.g., black African and white North American)
16. Prefer not to answer
17. You do not have an option that applies to me (please specify):

C5) In general, how would you rate your overall health?

1. Very poor
2. Poor
3. Average
4. Good
5. Excellent

C6) Approximately how long does it take you to drive from your place of residence to the PCFHC for an in-person appointment?

1. Less than 10 minutes
2. 10-29 minutes
3. 30-59 minutes
4. More than 60 minutes
5. N/A

C7) Do you have difficulty paying bills at the end of the month?

1. Yes
2. No
3. Sometimes
4. Prefer not to answer

C8) When you see or speak with nurses, physicians, physicians, specialists, and others in the healthcare system, in what language are you *most* comfortable?

1. English
2. French
3. I am most comfortable in another language (please specify):
